# Knowledge about tuberculosis among undergraduate health care students in 15 Italian universities: a cross-sectional study

**DOI:** 10.1186/1471-2458-14-970

**Published:** 2014-09-18

**Authors:** Maria Teresa Montagna, Christian Napoli, Silvio Tafuri, Antonella Agodi, Francesco Auxilia, Beatrice Casini, Maria Franca Coscia, Marcello Mario D’Errico, Margherita Ferrante, Angelo Fortunato, Cinzia Germinario, Domenico Martinelli, Giuseppe Michele Masanotti, Maria Fatima Massenti, Gabriele Messina, Paolo Montuori, Ida Mura, Giovanni Battista Orsi, Alessia Quaranta, Giovanni Sotgiu, Armando Stefanati, Stefano Tardivo, Maria Valeria Torregrossa, Anna Maria Tortorano, Licia Veronesi, Raffaele Zarrilli, Cesira Pasquarella

**Affiliations:** Department of Biomedical Science and Human Oncology—Hygiene Section, University of Bari Aldo Moro, Piazza Giulio Cesare 11, 70124 Bari, Italy; Department GF Ingrassia, University of Catania, Catania, Italy; Department of Biomedical Sciences for Health, Università degli Studi di Milano, Milan, Italy; Department of Translational Research, N.T.M.S., University of Pisa, Pisa, Italy; Department of Basic Medical Sciences, Neuroscience and Sense Organs—Hygiene Section, University of Bari Aldo Moro, Bari, Italy; Department of Biomedical Science and Public Health, Politecnica delle Marche, Ancona, Italy; Department of Interdisciplinary Medicine, University of Bari Aldo Moro, Taranto, Italy; Department of Medical and Surgical Sciences—Hygiene Section, University of Foggia, Foggia, Italy; Department of Experimental Medicine, University of Perugia, Perugia, Italy; Department of Health Promotion and Child Sciences ‘G. D’Alessandro’, University of Palermo, Palermo, Italy; Department of Molecular and Developmental Medicine, University of Siena, Siena, Italy; Department of Public Health, University of Napoli Federico II, Naples, Italy; Department of Biomedical Science—Hygiene Section, University of Sassari, Sassari, Italy; Department of Public Health, Sapienza University of Rome, Rome, Italy; Department of Biomedical Sciences—Epidemiology and Medical Statistics Unit, University of Sassari—Research, Medical Education and Professional Development Unit, AOU Sassari, Sassari, Italy; Department of Medical Sciences, University of Ferrara, Ferrara, Italy; Department of Public Health, University of Verona, Verona, Italy; Department of Biomedical, Biotechnological and Translational Sciences, University of Parma, Parma, Italy

**Keywords:** Knowledge, Tuberculosis, Undergraduate health care students

## Abstract

**Background:**

The Italian Study Group on Hospital Hygiene of the Italian Society of Hygiene, Preventive Medicine and Public Health conducted a multicentre survey aiming to evaluate undergraduate health care students’ knowledge of tuberculosis and tuberculosis control measures in Italy.

**Methods:**

In October 2012–June 2013, a sample of medical and nursing students from 15 Italian universities were enrolled on a voluntary basis and asked to complete an anonymous questionnaire investigating both general knowledge of tuberculosis (aetiology, clinical presentation, outcome, screening methods) and personal experiences and practices related to tuberculosis prevention. Data were analysed through multivariable regression using Stata software.

**Results:**

The sample consisted of 2,220 students in nursing (72.6%) and medicine (27.4%) courses. Our findings clearly showed that medical students had a better knowledge of tuberculosis than did nursing students.

Although the vast majority of the sample (up to 95%) answered questions about tuberculosis aetiology correctly, only 60% of the students gave the correct responses regarding clinical aspects and vaccine details. Overall, 66.9% of the students had been screened for tuberculosis, but less than 20% of those with a negative result on the tuberculin skin test were vaccinated. Multivariable regression analysis showed that age and type of study programme (nursing vs. medical course) were determinants of answering the questions correctly.

**Conclusions:**

Although our data showed sufficient knowledge on tuberculosis, this survey underlines the considerable need for improvement in knowledge about the disease, especially among nursing students. In light of the scientific recommendations concerning tuberculosis knowledge among students, progress of current health care curricula aimed to develop students’ skills in this field is needed.

## Background

In the last three decades, the emergence of HIV/AIDS as well as the appearance and spread of drug-resistant forms of tuberculosis (TB) have been associated with an increase in the TB rates in several low-, middle- and high-income countries [1, 2]. Together with HIV/AIDS and malaria, TB is one of the most significant causes of death worldwide, most frequently affecting men in their economically productive age groups [3, 4]. With a TB incidence rate considerably below 10 cases per 100,000 inhabitants over the last 10 years, Italy can be considered a low-burden country. Nevertheless, TB has increasingly become an illness affecting specific population subgroups; approximately 40% of reported TB cases in Italy involve foreign patients resident in Italy. Additionally, regional variation has been reported. Compared with Italy as a whole, TB rates are higher in the large cities of central and northern Italy, most likely because of the larger presence of foreigners or the more extensive commercial activity in these areas [5].

In 1993, the World Health Organization (WHO) declared TB a global public health emergency and supported national and international strategies to improve the care and control of the disease (i.e. Directly Observed Treatment, Short-course (DOTS) and the Stop TB Strategy) [6–8]. Although the TB mortality rate has decreased by 41% since 1990, *Mycobacterium tuberculosis* infection and related diseases remain a major global health issue. According to the latest estimates, there were 8.6 million new cases of TB and 1.3 million TB deaths in 2012 [4].

Findings from the global context indicate that a population’s knowledge of TB is crucial to facilitate the seeking of early medical care and avoidance of further *M. tuberculosis* transmission. Deficient knowledge often results in delays in TB diagnosis and treatment, increasing the risk of *M. tuberculosis* transmission and the development of multidrug-resistant TB (MDR-TB) across the world [4, 9, 10]. TB knowledge among undergraduate health care students is particularly important, because they may face significant exposure and, consequently, have the highest risk of infection or disease. Moreover, these individuals represent potential future physicians or leaders in the fight against TB, so it is important that they know how to control the disease appropriately [11, 12].

Based on this scientific background, the Italian Study Group on Hospital Hygiene (GISIO) of the Italian Society of Hygiene, Preventive Medicine and Public Health (SItI) promoted a multicentre survey that aimed to i) determine the level of knowledge of tuberculosis and its control measures among undergraduate health care students in Italy and ii) investigate personal experiences with practices to prevent *M. tuberculosis* infection.

## Methods

### Study design

Those Italian universities that were members of the GISIO group of the SItI and that offered medical and nursing degree courses were consulted. Overall, 15 universities located in urban areas participated in the survey on a voluntary bases. This study, carried out from October 2012 through June 2013, follows the principles of the World Medical Association Declaration of Helsinki and does not report any experiment on humans or human samples, nor research on identifiable human material and data.

All students took part on a voluntary basis and were not remunerated for their contribution. During the recruitment, potential participants were approached and provided with a detailed explanation of the objectives of the study. After participants’ verbal consent was obtained (as required by Italian privacy law), they were asked to complete an anonymous questionnaire.

### The questionnaire

The questionnaire consisted of multiple-choice questions divided into two sections comprising 13 and 7 questions, respectively: 1) general knowledge of TB disease, its aetiological agent, vaccine and screening methods and 2) personal experiences with and practices related to TB. The questionnaire also included questions about socio-demographic characteristics (i.e. age, gender, nationality, residence), location of the university and degree course.

To assess the accuracy of the questionnaire, an internal pre-validation procedure was carried out at the University of Bari Aldo Moro involving 20 fifth-year medical students and 10 second-year nursing students (Cronbach’s alpha = 0.83, indicating good internal consistency). This pilot phase allowed the improvement of the quality of several questions. Student participants in the pilot study were invited to complete the questionnaire in a time period of 20 minutes at the end of their lessons on hygiene.

### Data analysis

The information collected was entered into a database (File Maker Pro, 11.0v2, 2010) and analysed using Stata MP (11.2 for Mac, 2011). Here, the data are presented as percentages. The percentage distributions of the investigated variables were compared between medical students and nursing students using Chi-square tests. Continuous variables were summarised using means and standard deviations (SD) for variables that were distributed normally. Categorical variables were expressed as proportions. Univariable and multivariable logistic regression analysis was performed to evaluate the association between having correctly answered the questions about the disease, vaccine and screening methods (outcomes) and age, gender, degree course (medical vs. nursing) and tuberculin skin test (TST) result (determinants). The adjusted odds ratio (OR) and 95% confidence interval (CI) were calculated for each outcome. A p-value < 0.05 was regarded as statistically significant.

## Results and discussion

The study involved 2,220 Italian students enrolled in nursing (n = 1,611; 72.6%) and medicine (n = 609; 27.4%) courses. All of the participants correctly completed the questionnaire and were considered reliable for the analysis. No data were available with regard to the number of non-participants and how this could have affected the results of the survey. In our sample, the mean age was 22.5 ± 3.7 years, with the majority of the recruited individuals being female (66.4%).

Our findings showed a better knowledge of TB among medical students than among nursing students (Table 1). With regard to general information, the sample reported being aware that TB is an infectious disease caused by various strains of the *Mycobacterium* genus and more frequently by *M. tuberculosis* (94.7%), knew of the existence of MDR strains as a consequence of an inadequate therapy (78.5%) and were aware that TB does not affect only the lungs (75.8%). They also stated that the treatment of TB is problematic and requires the intake of a combination of antibiotics over a long period of time (75.1%), that the lethality rate of untreated active forms of TB is > 50% (66.5%) and that the most frequent *M. tuberculosis* infection outcome is latent infection (59.4%).

**Table 1:** **Percentage of enrolled students who correctly answered the tuberculosis-related questions, stratified by degree course**

Chosen answers	All students (n = 2,200)	Medical students (n = 609)	Nursing students (n = 1,611)	p
TB^1^ is caused by *M. tuberculosis*	94.7	99	93	<0.0001
MDR^2^ -isolates are a current problem for TB control	78.5	91.8	73.4	<0.0001
TB does not affect only the lungs	75.8	96	68.1	<0.0001
TB treatment is problematic and several drug must be prescribed for a long period	75.1	92.7	68.4	<0.0001
The lethality rate of untreated active TB is >50%	66.5	62.2	68.3	0.007
Several TB cases are asymptomatic	59.4	82	51.1	<0.0001
TB vaccine is currently available	87.4	90.4	86.3	0.009
TB vaccine is currently available, but it is not 100% effective	66.3	81.6	60.4	<0.0001
TB vaccine is composed by *Bacillus* Calmette Guerin	44.2	73.4	33.1	<0.0001
TST^3^ is aimed at detecting asymptomatic *M. tuberculosis* infection	88.0	90.6	87	0.02
TST is not a drug	69.7	88.3	62.6	<0.0001
TST is not a vaccine	68.6	87.3	61.5	<0.0001
TST is not a laboratory test	50.9	74.5	41.9	<0.0001

^1^TB = tuberculosis; ^2^MDR = multi-drug resistant; ^3^TST = tuberculin skin test.

Regarding knowledge of TB prevention, 87.4% of the student participants stated that they were aware of the existence of a vaccine, and 66.3% declared that this vaccine has a poor effectiveness. The characteristics of the vaccine were known to 44.2% of the sample.

Regarding the tuberculin skin test, the majority of those completing the questionnaires declared that the TST is helpful in diagnosing latent TB infection (88%), that it is not a drug for the treatment of *M. tuberculosis* infection (69.7%) and that it is not a vaccine (68.6%). However, of all study participants, 50.9% did not identify the TST’s usefulness for the isolation of *M. tuberculosis* from bronchial secretions.

In terms of personal experiences and practices related to TB, 66.9% of the enrolled students reported to have experienced—in the past—a screening test for the diagnosis of latent TB infection through the Mantoux test (93%), the Tine test (2.9%) or both tests (1.8%). The length of time before the administration of the questionnaire that the TST was performed was < 1 year for 71.7% of the sample, 1–5 years for 19.2% and > 5 years for 8.3%. For 12 students (0.8%), no previous TST was reported. Only 1,445 students reported the TST result; 39 (2.7%) had a positive result according to the guidelines of the Italian Ministry of Health [13], but none presented clinical or radiological evidence of active TB. Of the 1,406 TST-negative students, 281 (19.9%) were immunised with the Bacillus Calmette–Guérin (BCG) vaccine through public hygiene services (50.5%), through preventive medicine services (26.7%), by a general practitioner (6.4%), in anti-TB centres (5.3%) or in other locations (9.3%). For 1.8%, the structure where they were immunised was not reported.

The results of the uni- and multivariable logistic regression analyses performed to evaluate the associations between respondent characteristics and correct answers on the different items are reported in Tables 2 and 3. Regarding the questions concerning TB and its vaccine, providing the correct answers was associated with increasing age. In interpreting the results, it is useful to restate that the questionnaire was distributed at the end of the hygiene lessons. In Italy, the hygiene course is scheduled differently in different degree courses: it can be taught in the first, second or third year of the nursing course and in the fourth, fifth or sixth year of the medicine course. These differences in university planning might have introduced a bias in the results of the survey. The findings of the present study suggest that TB knowledge increases with age, as students develop more focused attitudes and behaviour. This possibility could explain the result from the nursing students but not from the medical students who had attended clinical wards and studied this topic in other subjects (e.g. infectious diseases, microbiology and pneumology).

**Table 2:** **Determinants associated with correct answers, estimated by univariable analysis**

Correct answers	Increasing age	Gender	To be a medical student	Previous TST
TB is an infectious disease caused by various strains of *Mycobacteria* genus, mainly *M. tuberculosis*	OR = 1.05	OR = 0.91	OR = 3.16	OR = 0.49
	95% CI = 0.98-1.12	95% CI = 0.62-1.36	95% CI = 3.28-17.1	95% CI = 0.31-0.77
	p = 0.12	p = 0.67	p < 0.0001	p = 0.002
TB does not affect only the lungs	OR = 1.09	OR = 1.15	OR = 2.44	OR = 0.41
	95% CI = 1.05-1.13	95% CI = 0.93-1.42	95% CI = 7.46-17.33	95% CI = 0.33-0.52
	p < 0.0001	p = 0.178	p < 0.0001	p < 0.0001
The most frequent outcome of the *M. tuberculosis* infection is the latent TB infection	OR = 1.11	OR = 1.27	OR = 4.36	OR = 0.79
	95% CI = 1.08-1.15	95% CI = 1.06-1.52	95% CI = 3.47-5.50	95% CI = 0.66-0.95
	p < 0.0001	p = 0.011	p < 0.0001	p = 0.013
Most of 50% of patients affected by active tuberculosis died	OR = 1.00	OR = 0.86	OR = 0.76	OR = 0.97
	95% CI = 0.98-1.02	95% CI = 0.71-1-03	95% CI = 0.63-0.93	95% CI = 0.81-1.17
	p = 0.92	p = 0.108	p = 0.007	p = 0.77
The treatment of the tuberculosis need the long-time use of antibiotics	OR = 1.13	OR = 1.46	OR = 5.87	OR = 0.40
	95% CI = 1.09-1.18	95% CI = 1.18-1.80	95% CI = 4.24-8.12	95% CI = 0.32-0.51
	p < 0.0001	p < 0.0001	p < 0.0001	p < 0.0001
The multidrug resistence of some strains of M. tuberculosis is an emergent concern	OR = 1.05	OR = 1.21	OR = 4.04	OR = 0.49
	95% CI = 1.01-1.08	95% CI = 0.97-1.50	95% CI = 2.96-5.51	95% CI = 0.39-0.62
	p = 0.005	p = 0.088	p < 0.0001	p < 0.0001
A vaccine against tuberculosis is available	OR = 1.00	OR = 1.04	OR = 1.50	OR = 1.20
	95% CI = 0.97-1.04	95% CI = 0.79-1.35	95% CI = 1.10-2.03	95% CI = 0.92-1.56
	p = 0.78	p = 0.79	p = 0.009	p = 0.171
The effectiveness of anti-tuberculosis vaccine is quite low	OR = 1.04	OR = 1.19	OR = 2.91	OR = 0.52
	95% CI = 1.01-1.06	95% CI = 0.98-1.43	95% CI = 2.31-3.66	95% CI = 0.43-0.63
	p = 0.007	p = 0.078	p < 0.0001	p < 0.0001
The TB vaccine is prepared with the *Bacillus* Calmette-Guérin	OR = 1.07	OR = 1.47	OR = 5.59	OR = 0.62
	95% CI = 1.94-1.09	95% CI = 1-24-1.77	95% CI = 4.54-6.89	95% CI = 0.52-0.75
	p < 0.0001	p < 0.0001	p < 0.0001	p < 0.0001
TST is not a vaccine	OR = 1.04	OR = 1.09	OR = 1.44	OR = 1.42
	95% CI = 1.01-1.09	95% CI = 0.82-1.42	95% CI = 1.06-1.96	95% CI = 1.09-1.85
	p = 0.04	p = 0.558	p = 0.021	p = 0.009
TST is not a laboratory test to detect *M. tuberculosis* in the sputum	OR = 1.07	OR = 1.14	OR = 4.32	OR = 0.66
	95% CI = 1.04-1.10	95% CI = 0.94-1.38	95% CI = 3.32-5.60	95% CI = 0.54-0.80
	p < 0.0001	p = 0.189	p < 0.0001	p < 0.0001
TST is not a drug for *M. tuberculosis* infection	OR = 1.05	OR = 1.22	OR = 4.05	OR = 0.71
	95% CI = 1.03-1.08	95% CI = 1.02-1.45	95% CI = 3.39-4.99	95% CI = 0.59-0.85
	p < 0.0001	p = 0.028	p < 0.0001	p < 0.0001

**Table 3:** **Determinants** ^**1**^ **associated with correct answers, estimated by multivariable regression**

Correct answers	Determinants	OR (95% CI)	p-value
TB is an infectious disease caused by various strains of *Mycobacteria* genus, mainly *M. tuberculosis*	Increasing age	1.50 (1.08 - 2.09)	0.02
TB does not affect only the lungs	To be a medical student	21.81 (2.89-164.38)	0.003
The most frequent outcome of the *M. tuberculosis* infection is the latent TB infection	To be a medical student	4.70 (1.83-12.04)	0.001
The TB vaccine is prepared with the *Bacillus* Calmette-Guérin	To be a medical student	13.43 (4.84-37.22)	0.001
TST is not a vaccine	Increasing age	1.10 (1.02-1.20)	0.02
TST is not a laboratory test to detect *M. tuberculosis* in the sputum	To be a medical student	2.19 (1.01-4.75)	0.047
TST is not a drug for *M. tuberculosis* infection	To be a medical student	2.90 (1.11-7.59)	0.03

^1^The investigated determinants are: age, gender, degree course (medical vs. nursing) and tuberculin skin test (TST).

Overall, our data highlight two main features: i) TB knowledge is sufficient among health care students (>60% of the enrolled students replied correctly to the questions asked) and ii) the level of knowledge is significantly higher among medical students than among nursing students. Regarding the first major finding, most student participants (up to 95%) chose the right answer to questions gauging general information on TB (aetiological agent, clinical forms, lethality and antibiotic resistance). Here, our data are not consistent with the results of other surveys, which have demonstrated different levels of TB knowledge [14–17]. Moreover, other studies have reported that only one-third of the participants provided correct answers to questions about disease transmission [17], and misconceptions among health professionals concerning TB transmission and therapy have been described [18, 19].

Regarding knowledge of asymptomatic cases of TB infection, our study yielded less positive results: only 60% of the total sample provided the correct answer. Considering the role of asymptomatic cases in the epidemiology of TB disease, these data are cause for concern. Moreover, only 44.2% of participants in this study knew the TB vaccine composition, and only two-thirds of the sample were aware that the current vaccine is not 100% effective. Given that the TB vaccine is a key preventive measure against some of the most severe manifestations of the primary infection, such as hematogenous dissemination and meningoencephalitis, and that there is no strong evidence of the vaccine’s efficacy against the pulmonary form of TB, these data appear critical.

Concerning the TST, the frequency of correct answers was high only for those questions about the detection of asymptomatic infected individuals. For other questions (i.e. TST is a vaccine, laboratory test or drug), more than 30% of the participants answered incorrectly. These data are consistent with the results that have been reported from some other authors [16].

Regarding the second main finding, the medical students in our sample provided correct answers in most cases (from 62.2% to 99%), and their level of knowledge was significantly higher than that of the nursing students. Although this result is consistent with the different level of knowledge required in these degree courses, poor knowledge of TB may raise crucial questions for health care workers employed in high-risk clinical areas in terms of perceptions of occupational risk and patient safety. Health care workers, especially nurses, can be exposed to *M. tuberculosis* during their routine clinical activities. Additionally, they can play an important role in patients’ symptom identification, education of patients and families about TB diagnosis and treatment, supporting patients’ adherence to their therapeutic regimens, the effective utilisation of health care services and the adequate management of adverse drug events [20]. Therefore, when these members of the health care system exhibit poor knowledge of TB, the outcomes of TB treatment and of preventive programmes may be at risk of failure. Thus, it is crucial to plan and implement strategies and policies to improve the knowledge of health care team members towards TB, including the capacity to integrate knowledge and good practices [21, 22].

## Conclusions

Our study found sufficient TB knowledge in a sample of Italian health care students. Nevertheless, we consider it is necessary to improve knowledge about TB, especially among nursing students. TB knowledge among health care undergraduates is important, because these students could be exposed to the *Mycobacterium* strains during their training activities or when they are employed in private and public health care settings. Consequently, consistent with the conclusions of other authors [16, 23, 24], we suggest upgrading the current health care curricula. The training provided through courses of study should provide the scientific basis necessary to achieve an appropriate level of professional autonomy. Students’ skills in this field can be developed by promoting an integrated, multidisciplinary study programme focused on problem-oriented learning and active learning strategies (e.g. seminars, computer simulations, etc.).
